# Detailed Analysis of Dorsal-Ventral Gradients of Gene Expression in the Hippocampus of Adult Rats

**DOI:** 10.3390/ijms23179948

**Published:** 2022-09-01

**Authors:** Alexander Beletskiy, Ekaterina Positselskaya, Aliya Kh. Vinarskaya, Yulia S. Spivak, Yulia V. Dobryakova, Iliya Tyulenev, Vladimir A. Markevich, Alexey P. Bolshakov

**Affiliations:** Institute of Higher Nervous Activity and Neurophysiology, Russian Academy of Sciences, 117485 Moscow, Russia

**Keywords:** dorsal hippocampus, ventral hippocampus, RNAseq, splicing, transposable elements, ribosomal RNA depletion

## Abstract

We performed RNA sequencing of the dorsal and ventral parts of the hippocampus and compared it with previously published data to determine the differences in the dorsoventral gradients of gene expression that may result from biological or technical variability. Our data suggest that the dorsal and ventral parts of the hippocampus differ in the expression of genes related to signaling pathways mediated by classical neurotransmitters (glutamate, GABA, monoamines, etc.) as well as peptide and Wnt ligands. These hippocampal parts also diverge in the expression of axon-guiding molecules (both receptors and ligands) and splice isoforms of genes associated with intercellular signaling and cell adhesion. Furthermore, analysis of differential expressions of genes specific for astrocytes, microglia, oligodendrocytes, and vascular cells suggests that non-neuronal cells may also differ in the characteristics between hippocampal parts. Analysis of expression of transposable elements showed that depletion of ribosomal RNA strongly increased the representation of transposable elements in the RNA libraries and helped to detect a weak predominance of expression of these elements in the ventral hippocampus. Our data revealed new molecular dimensions of functional differences between the dorsal and ventral hippocampus and points to possible cascades that may be involved in the longitudinal organization of the hippocampus.

## 1. Introduction

The first histological studies have shown that the hippocampus is a highly organized brain structure that includes the CA1-CA3 areas and the dentate gyrus. At first glance, histological uniformity along the longitudinal axis of the hippocampus seems to point to functional homogeneity of hippocampal cells located in the dorsal and ventral parts of the hippocampus. However, electrophysiological studies performed in the 1960s showed that EEG activity recorded in the dorsal and ventral parts of the hippocampus as well as connections formed by these two hippocampal parts strongly differ [1,2,3]. Later behavioral studies showed that selective lesioning of dorsal and ventral parts of the hippocampus induces different behavioral effects [4,5]. Analysis of connectivity also showed that the dorsal and ventral parts of the hippocampus send and receive projections from different brain areas [6]. Moreover, a recent study points to a different transcriptional response of the dorsal and ventral hippocampus in male and female mice to chronic corticosterone administration [7]. The results of studies on the analysis of the functions of the dorsal and ventral hippocampus under various conditions were summarized in numerous reviews [6,8,9,10,11,12].

Thus, physiological and behavioral studies laid the basis for the idea that, despite similar histological organization, the dorsal and ventral parts of the hippocampus have different functions and this difference may be related to the differences in the characteristics of the cells in these hippocampal parts. Recent studies employing next generation sequencing have helped to reveal the major sets of genes that determine the specific features of the cells in the dorsal and ventral hippocampus [13,14,15]. Moreover, it was shown that there is a subset of genes whose gradient of expression between the dorsal and ventral parts of the hippocampus remains stable from postnatal day 14 till postnatal day 45, whereas the expression of other genes changes with animal maturation [14]. However, the mentioned studies used quite a low coverage (7–15 million reads [13,14]), which is enough for detection of only highly expressed genes with strong differences between the groups [16]. In addition, previous studies used polyA-enrichment for the preparation of sequencing libraries [14] or were focused on mRNA expressed selectively in hippocampal neurons [15]. Low coverage, selective analysis of some subgroups of mRNA, and small number of samples in previous studies did not allow an analysis of the variability of dorsoventral gradient among animals and performance of detailed analysis of possible dorsoventral gradients of splice isoforms between hippocampal parts and analysis of expression of transposable elements, which are also expressed in the brain cells [17,18]. Furthermore, it was shown that cell subpopulations in the brain have specific transcriptomic signatures [19], however, the question about differences in the characteristics of non-neuronal cells between the dorsal and ventral parts of the hippocampus has not been addressed so far. It also is still unclear whether the gradient of gene expression between the dorsal and ventral parts of the hippocampus is stable among animals of similar age and whether the technical approaches can affect the analysis outcome. Therefore, the aim of our study was to evaluate the dorsoventral gradients of gene expression using RNAseq data obtained in a larger set of samples with a higher coverage after polyA-enrichment or depletion of ribosomal RNA and to compare it with data from previous studies.

## 2. Results

**Figure 1:**
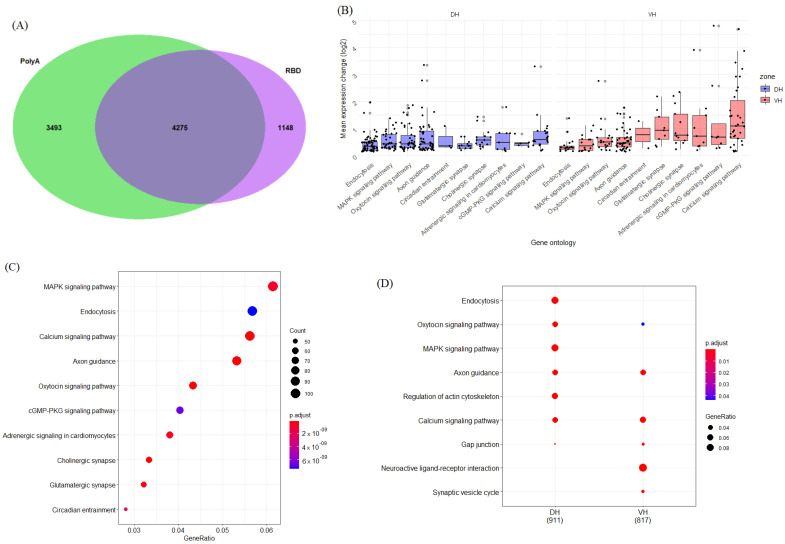
(**A**) Intersection between genes differentially expressed (dorsal vs. ventral hippocampus (DH vs. VH)) in PolyA and RBD datasets. (**B**) Top 10 DH- and VH-dominant (blue, red) gene ontology categories for PolyA/RBD intersection gene set. *X* axis represents ontology category; *Y* axis, log_2_ fold change, averaged across all genes in the group. (**C**) Plot showing top 10 most significant KEGG categories for PolyA/RBD intersection gene set (4275). (**D**) Plot showing statistical comparison of KEGG category enrichment in Ventral (VH) vs. Dorsal (DH) gene lists.

First of all, we analyzed the number of genes that are differentially expressed (DE) between the dorsal and ventral parts of the hippocampus in a polyA-enriched fraction (*n* = 7 animals) and samples that underwent rRNA depletion (RBD (*n* = 3 animals). We found that number of DE genes in polyA-enriched samples was 7768 and 5423 genes in RBD samples (Figure 1A). Only 4275 genes had the same character of differential expression in both groups of samples. When we categorized these genes, we found that the differences between the dorsal and ventral parts of the hippocampus were associated with groups of genes related to “MAPK signaling pathways”, “Endocytosis”, “Calcium signaling pathway”, “Axon guidance”, etc., (Figure 1B–D). The DE genes in these groups did not have uniform predominance in one part of the hippocampus versus another but rather included subgroups of genes that were predominantly expressed in either the dorsal or ventral part of the hippocampus (Figure 1C,D). Note, however, that the expression of genes related to “Neuroactive ligand-receptor interaction” was shifted to the ventral hippocampus (Figure 1D), which, as we will show below, reflects a higher expression of genes encoding receptors of major CNS neuromediators in this hippocampal part.

Our next step was to categorize common DE genes in polyA-enriched and RBD samples in a more detailed way related to the involvement of the products of DE genes in intercellular communication. For the sake of simplicity in the figures below, we showed the 10 top genes from KEGG categories with the highest expression difference between the hippocampal parts and the rest of the genes from the same category are presented in the supplementary figures.

### 2.1. Function-Related Differences in Gene Expression between the Dorsal and Ventral Hippocampus

#### 2.1.1. Genes Related to Classical Neurotransmitters

**Figure 2:**
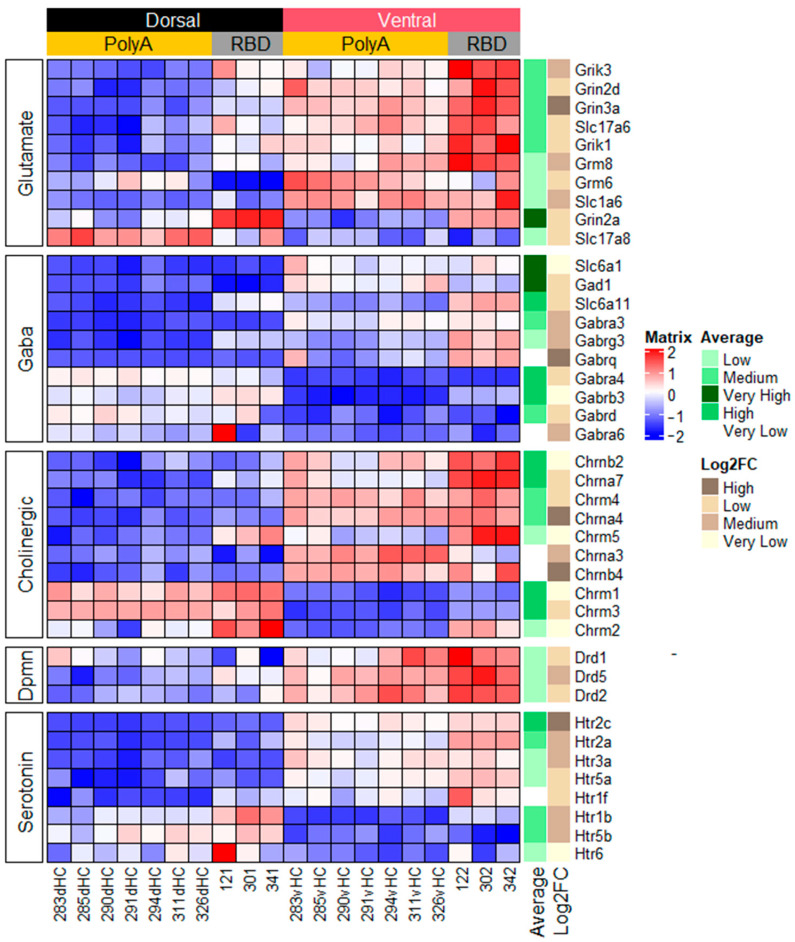
Heatmap showing 10 most DE genes for 5 KEGG categories, related to classical neurotransmitter signaling. Colors represent variation around the mean, red color signifies higher expression values. Average (gradation of green) represents mean expression across all datasets. We discretized average values as follows: Very Low, <50 normalized counts; Low, 50–300; Middle, 300–1000; High, 1000–5000; Very High, >5000. Log_2_FC (gradations of brown) is absolute log_2_ fold change with sign depending on heatmap color. Log_2_FC values were discretized as follows: Very Low, <0.5; Low, 0.5–1; Middle, 1–2; High, >2. Dpmn, dopamine.

The first categorized genes were related to signaling via classical neurotransmitters and included the genes encoding the receptors and transporters of these neurotransmitters. Glutamate is the major excitatory neurotransmitter in the CNS and our analysis showed that 18 genes related to glutamate signaling were differentially expressed between the dorsal and ventral hippocampus (Figure 2 and Figure S1) and the majority of them were subunits of NMDA, AMPA, and kainate receptors as well as glutamate metabotropic receptors.

Analysis of the DE genes related to inhibitory GABAergic transmission also revealed that the cells in the different parts of the hippocampus may express different subunits of GABAA receptors and transporters related to GABA signaling.

Genes related to cholinergic, dopaminergic, noradrenergic, and serotonergic signaling were also differentially expressed between the hippocampal parts (Figure 2 and Figure S1). The number of DE genes related purinergic and histaminergic systems was less numerous. Only expression of the genes of the adenosine receptor *adora1* and histamine receptor *hrh3* was shifted to one of hippocampal parts; *adora1* was higher in the dorsal hippocampus whereas *hrh3*, which is predominantly expressed in neurons [20], was predominant in the ventral hippocampus.

#### 2.1.2. Genes Related to Peptide or Protein Ligands and Their Receptors

**Figure 3:**
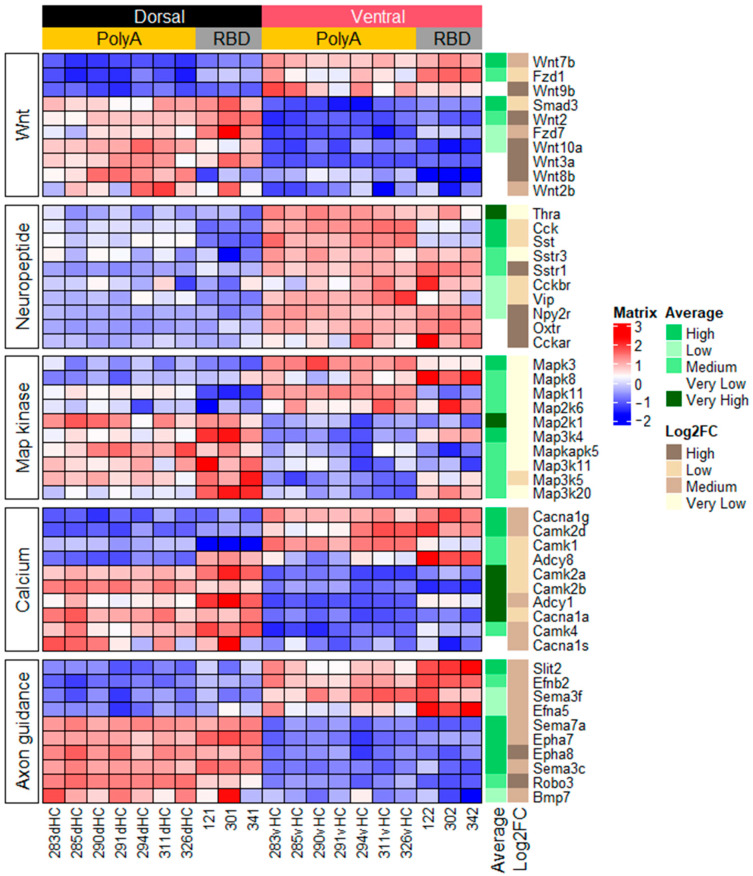
Heatmap showing 10 most DE genes for 5 KEGG categories related to calcium signaling and axon guidance cues. Colors represent variation around the mean, red color signifies higher expression values. All designations are identical to Figure 2.

Analysis of DE genes related to protein and peptide intercellular signaling showed that the dorsal and ventral hippocampal parts differed in the expression of ligands and receptors involved in wnt signaling (Figure 3). Other dorsoventral differences also included higher expression in the ventral hippocampus of receptors of somatostatin (sst), cholecystokinin (cck), neuropeptide Y, oxytocin, and thyroid hormones as well as signaling peptides predominantly expressed in hippocampal interneurons such vasoactive intestinal peptide (vip) and *sst*.

The general picture of the predominant expression of the receptors of various transmitters as well as their modulators in one of hippocampal parts is summarized in Figure S2. Note that sometimes the predominant expression of the receptors in one hippocampal part was associated with a higher expression of peptide ligands of the same receptors in the same hippocampal part (*cck* and *cckr*, *sst* and *sstr*, *tac1/4* and *tacr1*, *lynx*, and nicotinic acetylcholine receptors). However, for some ligand-receptor pairs, the relationships of the expression were opposite, i.e., expression of the receptor was higher in one hippocampal part whereas expression of the ligand was higher in another part (*nts* and *ntsr1*, *gal* and *galr*, *tac3* (*tac2* in mice) and *tacr3*, *trh* and *trhr*, *agt*, and *mas1*).

The next clear subgroup of genes whose expression differed between the dorsal and ventral hippocampal parts consisted of genes related to axon guidance cues and included genes predominantly encoding transmembrane proteins responsible for cell–cell interaction such as Netrins, Slits and their receptors (Robo), Ephrins and their receptors, and Semaphorins and their receptors (Plexins and Neuropilins) (Figure 3 and Figure S1). A diagram summarizing the relationship between the specific expression of proteins and their location in the hippocampus is shown in Figure S3. One specific detail may be seen in this diagram as well as in the data in Figure 3, viz., the expression of protein ligands (ephrins, Slits, some Semaphorins, and some Netrins) is predominantly shifted to the ventral hippocampus whereas the expression of their receptors (Eph receptors, Robos, and Plexins) is predominant in the dorsal hippocampus.

#### 2.1.3. Genes Related to Other Signaling Pathways

Next, we analyzed DE genes whose products are involved in intracellular calcium-mediated signaling and MAP kinase cascades (Figure 3 and Figure S1). The first group of genes related to calcium signaling included subunits of voltage-gated calcium channels (cacn genes). Interestingly, this group included only the alpha1 subunits of voltage gated channels despite the fact that practically all subunits were expressed in both hippocampal parts (except cacng1). The second group included genes encoding adenylate cyclases (adcy genes) activated by calcium (adcy1, adcy3, and adcy8) and adcy9 activated by G-proteins. Note that adcy3 and adcy8 were expressed at a higher level in the ventral hippocampus and the expression of the other two was higher in the dorsal part. Since the glutamate signaling pathway closely intersects with the G-protein- and Ca^2+^-signaling pathways, we summarized the data on the expression of genes associated with these signaling pathways on the diagram shown in Figure S4. As for the MAP kinase-related genes, this subgroup included 17 genes (Figure 3 and Figure S1) and the diagram summarizing their role in the MAP kinase cascades is shown in Figure S5. One salient detail that may be seen from this diagram is that tyrosine phosphatases related to the PTP and MKP families, which can inhibit ERK and JNK kinases, were predominantly expressed in the dorsal hippocampus whereas the expression of their targets ERK and JNK was shifted to the ventral hippocampus. The latter may point to the potentially higher role played by the ERK- and JNK-mediated pathways in the cells of the ventral hippocampus or a higher control over ERK and JNK by the mentioned phosphatases in the dorsal hippocampus.

### 2.2. Cell-Specific Differences in Gene Expression between the Dorsal and Ventral Hippocampus

**Figure 4:**
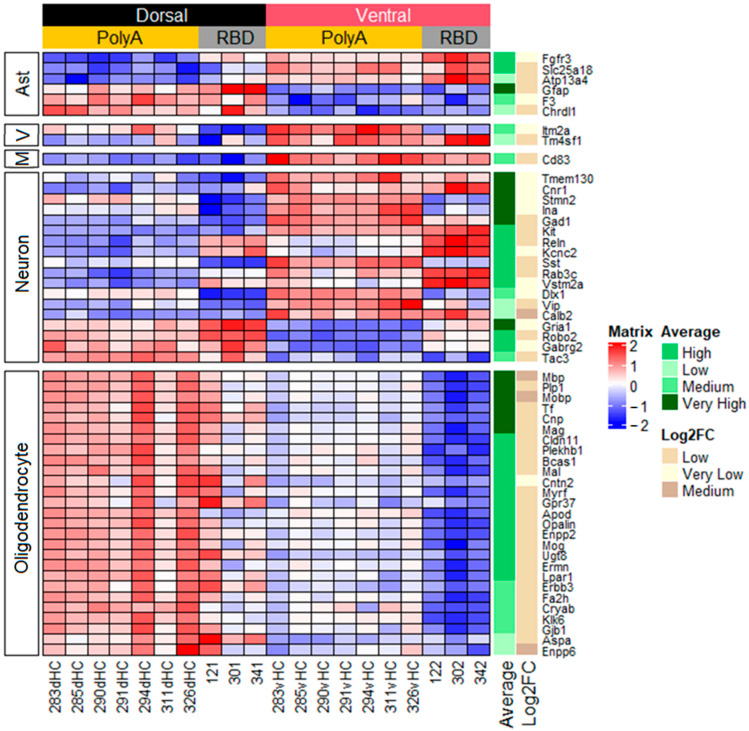
Heatmap showing DE genes, present in lists of top 30 most expressed cell-type-specific genes from [19]. Ast, astrocytes; V, vascular cells; M, microglia. All other designations are identical to Figure 2.

The classification of genes may be performed not only on the basis of the functional role played by the gene product but also on the basis of cell subpopulations where the DE genes are predominantly expressed [19]. Therefore, we performed classification genes that are selectively expressed in one of the following cell subpopulations: astrocytes, microglia, vascular cells, neurons, and oligodendrocytes. Among these selectively expressed genes, we took 30 genes that are highly selectively expressed in a given cell subpopulation and using this classification identified subpopulation-selective genes among genes that were differentially expressed between the dorsal and ventral hippocampus. We found that only six genes related to astrocytes were differentially expressed between the hippocampal parts (Figure 4). Analysis of the DE genes specific for microglia showed that cd83 was the single gene differentially expressed between the dorsal and ventral hippocampal parts and its mRNA level was higher in the ventral hippocampus. As for genes encoding proteins selectively expressed in vascular cells, the expression of itm2a and tm4sf1 was upregulated in the ventral hippocampus. The neuron-specific genes included 18 genes and some of them may be united in the group of interneurons-specific genes (*sst*, *vip*, *cnr1*, *gad1*, *reln*, *dlx1*, *tac3*). The largest group (20 genes) of cell-specific genes consisted of oligodendrocyte-specific genes and their expression was increased in the dorsal compared to the ventral hippocampus.

### 2.3. Differences in the Dorsoventral Gradient between Datasets Are Related to Genes Expressed in All Cell Types

The results presented in Figure 1 suggest that a large group of genes were differentially expressed between the dorsal and ventral hippocampus; however, they did not coincide between the polyA-enriched and RBD samples. Therefore, we decided to analyze the groups of DE genes that presented only in polyA and RBD samples (3493 and 1148 genes, respectively). Despite a large number of genes in each case, classification using KEGG categories revealed relatively small number of gene groups. The RBD-specific groups of DE genes included genes related to inositol signaling and ribosome proteins (Figure S6A,B). In the polyA-enriched samples, the groups of genes that were differentially expressed between the dorsal and ventral hippocampus included the genes associated with ribosomal proteins, oxidative phosphorylation, transport, ubiquitin signaling, and spliceosome (Figure S6C,D). Note that in both groups of samples, the genes had a medium to very high average expression level and a very low differential expression or very low basal expression and medium Log_2_FC. These characteristics of expression suggest that addition of a single sample with deviant expression in one hippocampal part can easily lead to the exclusion of this gene from the set of genes with a dorsoventral gradient of expression.

### 2.4. Genes with Stable Dorsoventral Gradients of Expression

**Figure 5:**
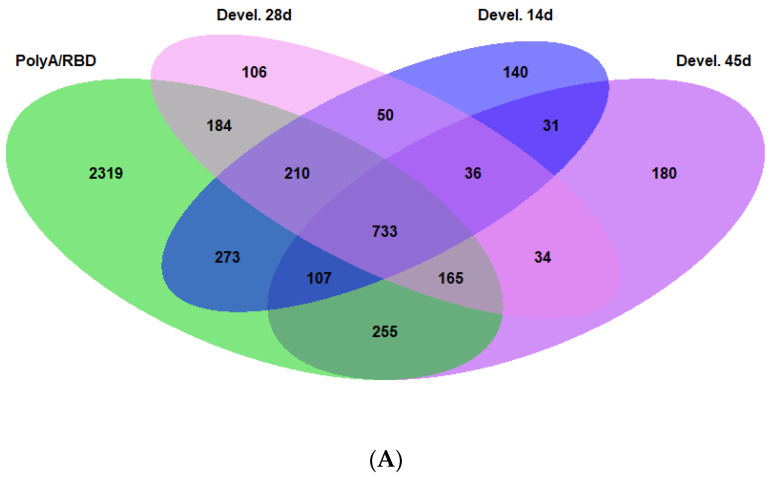

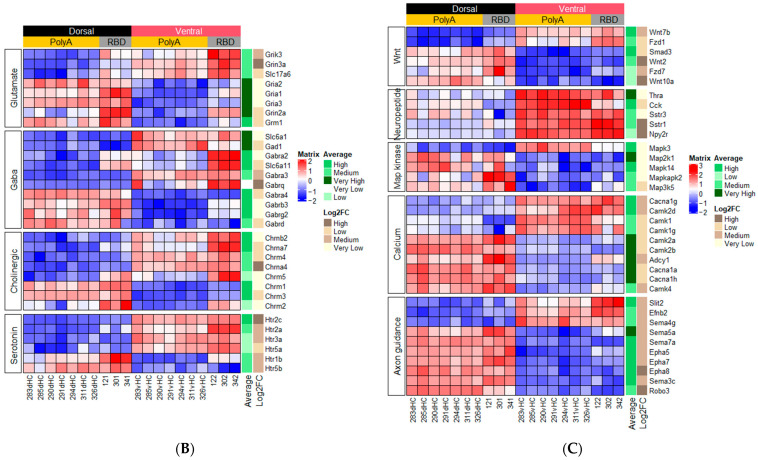
(**A**) Intersection between genes differentially expressed (VH vs. DH) in polyA/RBD conserved gene set (4275) and hippocampal development dataset from [14]. (**B**,**C**) Heatmaps for genes from intersection between our dataset and datasets for P45 from [14].

Our next step was to analyze whether the dorsoventral gradients of expression described above were stable and did not change during postnatal ontogeny and were independent of animal origin and sequencing approaches. To this aim, we took data from previous publications [13,14], annotated them, and analyzed the intersection of the dorsoventral gradient of gene expression between our samples and the samples taken from the mentioned studies. We found that the number of genes whose dorsoventral gradient of expression was the same in our samples and the samples from [14] at the age of 45 days (P45) was 1260 (Figure 5). To identify ontogenetically stable DE genes, we intersected them with DE genes at P28 and P14 and the number reduced to 733 (Figure 5A). We also categorized these ontogenetically stable genes using the above principles and found that the number DE genes related to glutamate, GABA, and other signaling pathways reduced (Figure 5B,C).

**Figure 6:**
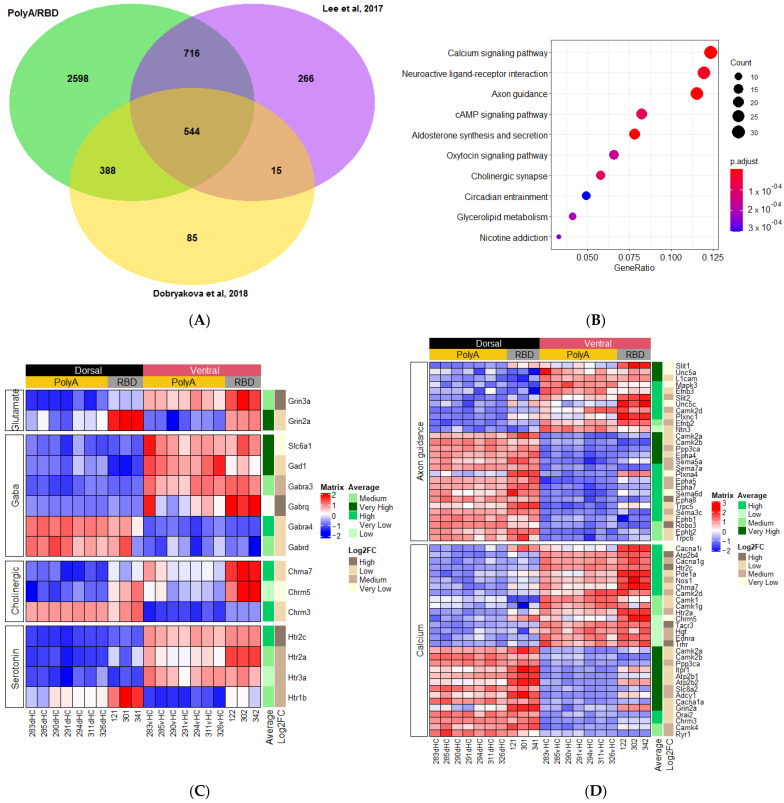
(**A**) Intersection between genes differentially expressed (VH vs. DH) in PolyA/RBD conserved gene set (4275) (green), our previously published data [13] (yellow), and hippocampal development dataset from [14] (purple). (**B**) KEGG plot for 544 genes that fall into intersection. (**C**,**D**) Heatmaps showing neurotransmitter and calcium/axon guidance KEGG category genes from 544 ‘core’ set.

We also analyzed the intersection of the DE genes in our samples and the samples taken from adult animals in [13,14]. We found that the number of DE genes was 544, i.e., the variability of technical conditions and animal strains between the studies strongly reduced the number of DE genes between the hippocampal parts (Figure 6A). Further categorization of the DE genes also revealed a strong reduction or even disappearance of some groups of genes related to the aforementioned signaling pathways (Figure 6B–D). However, the last comparison of the DE genes between the studies showed that there was still a set of genes that were differentially expressed between the dorsal and ventral hippocampus which form some sort of “core” genes whose expression is stably different in adult rats.

### 2.5. Differential Alternative Splicing between the Dorsal and Ventral Hippocampus

**Figure 7:**
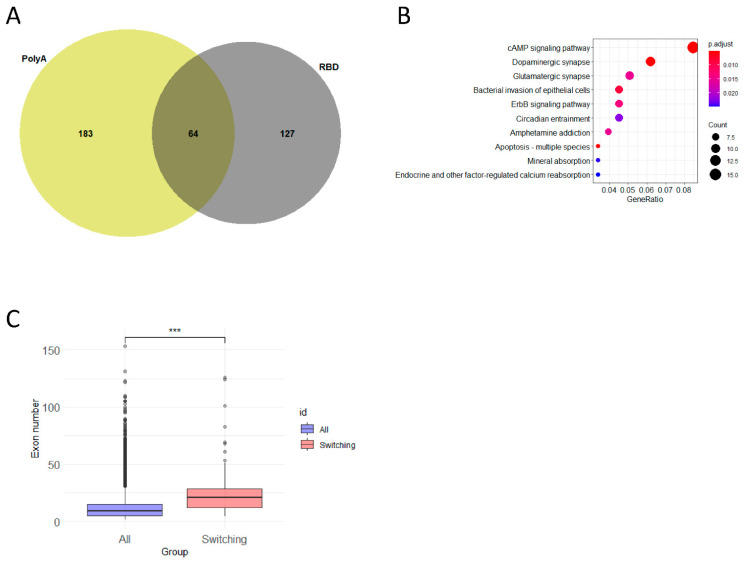
(**A**) Intersection between genes undergoing differential transcript usage (DTU) in PolyA (VH vs. DH) and RBD (VH vs. DH) datasets. (**B**) Top 10 KEGG categories for genes that fell into intersection from (**A**). (**C**) Comparison of mean exon numbers between DTU and non-DTU genes. ***, result is significant with *p* < 0.05 (Mann-Whitney test).

Our next aim was to analyze whether mRNA transcripts expressed in the dorsal and ventral hippocampus may represent different splice isoforms of the same mRNA. To this aim, we used the DEXseq-stageR pipeline [21]. We found that among the polyA-enriched and RBD samples, the number of differentially spliced (DS) mRNAs was different and only 64 genes had a similar character of dorsoventral gradient of splicing in both types of samples (Figure 7A). Differentially spliced mRNAs were predominantly related to the cAMP signaling pathway, and the dopaminergic and glutamatergic synapses (Figure 7B) had a higher number of exons than all the other annotated genes (Figure 7C). The problem of classification shown in Figure 7B is that it is meaningless from a physiological viewpoint. Therefore, since the number of DS genes was relatively small, we analyzed these genes in greater detail and found that the DS genes between the dorsal and ventral hippocampus included genes encoding cytoskeleton-related proteins (*ank2*, *map2*, *pclo*, *mprip*, *klc1*, *fmn1*, *kif1b*, *cyfip1*, *plec*) and membrane-bound proteins related to the formation of synapses (*grm1*, *gria3*, *rims2*, *shank2*, *bin1*, *mpp6*) and cell adhesion or extracellular matrices (*adgrl1*, *ncam2*, *cadm1*, *tspan9*, *nrcam*, *rtn3*, *rtn4*, *itih3*, *nrxn1*, *nrxn2*). Detailed analysis of differential splicing showed that the difference between the dorsal and ventral parts of the hippocampus consisted of the predominant expression of shorter or longer splice variants or exon skipping in one of the hippocampal parts (Figure S7). It should be noted that, in the case of DS genes, we found a strong dependence of the detected splicing forms on the approach used for the preparation of the RNAseq libraries. This may be seen by the example of several of the genes presented in Figure S7. For example, *nrxn1* expression in the ventral hippocampus was characterized by predominance of 5′ exons in the RBD samples whereas in the polyA-enriched samples 3′ exons were more numerous. Similarly, in the dorsal hippocampus, nrxn1 exons closer to 5′-end were enriched in the RBD samples, whereas 3′-associated exons were more numerous in the polyA-enriched fraction. However, despite this difference in the coverage of exons, it may be noted that, in both the RBD and polyA samples, the dorsal and ventral areas of the hippocampus differed in the splice variants. The dorsal hippocampus in the polyA samples had a higher content of shorter *nrxn1* variants compared to the ventral hippocampus (both lacked the 16th exon bin). In the RBD samples, the ventral hippocampus also contained longer variants than the dorsal hippocampus, however, they both contained the 16th exon bin, pointing to possibility that there may be a transcript related to the nrxn1 gene, which terminates at the 16th exon bin and is not polyadenylated. A similar character of DS between the RBD and polyA samples was found for other DS genes (Figure S7).

### 2.6. Analysis of Differential Expression of Transposable Elements

**Figure 8:**
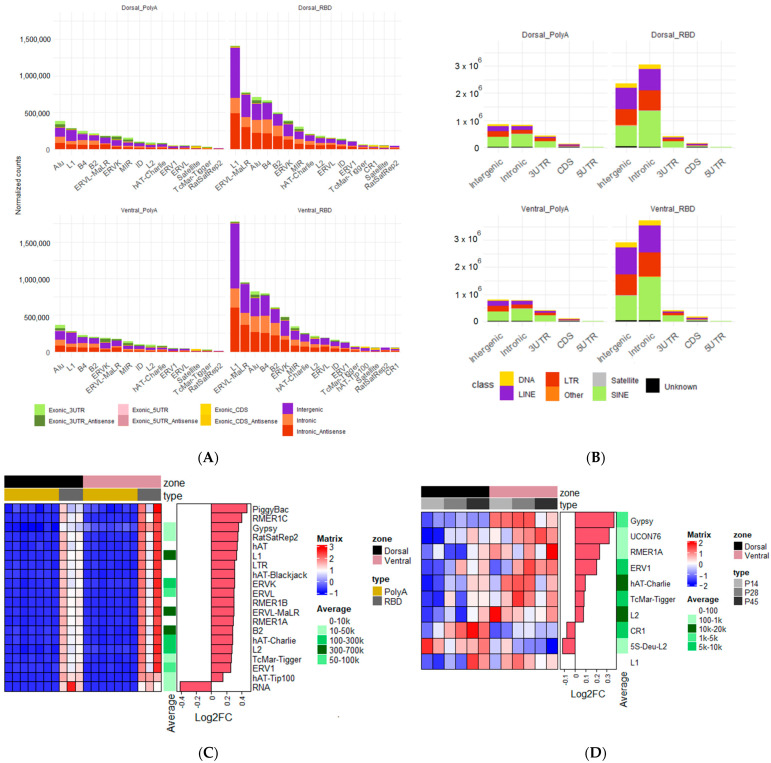
(**A**) Normalized read count per repeat family, summarized by number of different genic/intergenic positioned loci in each. (**B**) Normalized read count per genic/genomic position, summarized by repeat class. (**C**) Heatmap showing z-scored repeat-expression difference between four datasets (left), read number averaged across all datasets (middle), and relevant log_2_ fold change (right). All changes were significant for RBD dataset with *p*. adj < 0.1. (**D**) Heatmap with similar measurements for developmental dataset from [14].

Finally, we decided to assess the ventral/dorsal differences in the expression of various transposable elements (TE), which are known to be abundant in the adult brain [17,18]. To achieve this goal, we re-mapped all of the reads from the PolyA and RBD samples and quantified their mappings to genomic repeats, present in the Repeatmasker annotation, with respect to their position at the most proximal genes (Figure 8A).

The main difference to note was related not to the hippocampus itself but to the cDNA-library preparation method. The non-polyA filtered (RBD) samples preserved a much higher amount of repeat reads with the most significant proportion of them mapping to the L1 family (Figure 8A), while the polyA samples contained a smaller read number and had Alu as its most expressed group (Figure 8A). All expressed TE from both datasets were predominantly located in introns or were intergenic (Figure 8B), though this difference suddenly shifted to a dominance of 3′UTR loci, if we limited the total number of repeats per family to only the 1000 most expressed (Figure S8A). We also observed the unexpected absence of TE-loci contained in genic 5′UTRs numbering less than 1% for all datasets and groups (Figure 8B).

Given the somewhat noticeable differences between the dorsal and ventral areas of the hippocampus in the RBD datasets (Figure 8A), we also performed a differential expression analysis on aggregated TE family reads and indeed found this difference to be significant for the RBD data (*p*. adj. < 0.1) with a slightly higher expression in the ventral hippocampus for most TE types (log2FC ~0.2–0.3) and present in two out of three samples (Figure 8C). To check whether these results were biased toward our data, we repeated a similar analysis with the hippocampal development dataset [14] and also found a significant dorsal/ventral TE expression difference, with some of the same TE families being more abundant in the ventral hippocampus across different developmental stages (Figure 8D). We also observed the same tendency of 3′UTR-positioned TE loci predominance in the development dataset (Figure S9), which, given its quite low coverage, could suggest their earliest read saturation during sequencing.

## 3. Discussion

Our study is a continuation of previous studies [13,14,15] that were focused on the analysis of the molecular differences between the dorsal and ventral parts of the hippocampus. The main advantage of our study compared to the previous studies is that we used a significantly higher coverage and larger number of animals in the groups. In addition, we also included a group of samples where, instead of standard polyA-enrichment [14], depletion of ribosomal RNA was performed, which helped us to detect additional features of the dorsoventral differences. Our analysis also included an estimation of possible cell-specific mRNAs, differential splicing, and expression of various transposable elements.

### 3.1. Technical Issues

Our comparison of the dorsoventral differences in gene expression between the datasets from different studies suggests that there was some dissimilarity between our data and data of previous studies [13,14] and the strongest difference appeared with the dataset from the study of the developmental analysis of dorsoventral gradients [14]. Presumably, a very low coverage and small number of animals (two rats in one group) could lead to the detection of irrelevant dorsoventral differences, although the contribution of biological variability and changes related to the aging of animals (our animals were aged 3 months compared to 45 days in [14]) cannot be excluded. It should be noted that the RNAseq analysis in our previous study [13] was performed in the hippocampi of animals subjected to surgery (intracerebroventricular injection of saline) and a series of behavioral tests (Morris water maze, open field, T-maze, and passive avoidance). It may be expected that this battery of behavioral tests could affect the functioning of both hippocampal parts in a different fashion and shift the dorsoventral gradients of gene expression compared to resting animals. However, less than 10% of DE genes in [13] did not coincide with the DE genes in the current study suggesting that the detected differences between the hippocampal parts are unlikely to be sensitive to stresses.

It is worth noting that our study was focused on the analysis of the differential expression of RNA between the hippocampal parts and may be considered non-relevant from a physiological viewpoint since the major functions are performed by proteins, not RNA. However, in our study, we were not focused on the analysis of the processes that are induced by some stimuli but rather analyzed the steady state when no additional stimuli were provided to the animals to provoke an additional transcriptional or translational response. The latter is important since, under the conditions of physiological balance, the differences in the level of RNA are likely to be directly related to the differences in protein level and the functions performed by these proteins.

### 3.2. Functional Importance of Dorsoventral Gradients

Our analysis shows that many axon-guiding ligands and their receptors as well as genes associated with wnt signaling were expressed in both the dorsal and ventral hippocampus of adult rats. Moreover, there was a gradient of the expression of some of these genes between the dorsal and ventral hippocampus. The majority of these axon-guiding and wnt-related molecules are critical in early ontogeny during the formation of synapses within and between brain structures. The fact that their expression did not cease after animal maturation points to other important roles played by these molecules in the functioning of hippocampal cells. In adult animals, some semaphorins are critical for hippocampal synaptic plasticity whereas others are proposed to be critical for neurogenesis and axon guidance under some conditions [22]. The problem is that sema5a and sema7a, whose roles are predominantly studied in neurogenesis, are expressed not only in the dentate gyrus but also in the CA3 and CA1 areas [15], which are not related to neurogenesis. A similar situation may be seen for ephrins, robo-slit, and wnt signaling, where their roles are established during synapse formation in early ontogeny and are practically unknown in adult animals [23,24]. In addition to the mentioned dorsoventral gradients of axon-guiding molecules, we also found differences between the hippocampal parts in the splicing of mRNA encoding molecules involved in intercellular interaction and the formation of synapses such as neurexins, ank2, pclo, shank2, nrcam, etc. The latter implies that the formation and functioning of “typical” synapses in the dorsal and ventral areas of the hippocampus may have substantially different structural bases and, as a consequence, specific regulation. Our data also suggest that the specific characteristics of the hippocampal parts comes not only from the composition of the intracellular and synaptic proteins but also from the predominance of the specific receptors of classical mediators in neurons of a given hippocampal part. Thus, it may be concluded that the convergence of both mediator combination and specific cell interaction molecules provides basis for the uniqueness of cell–environment interaction in the dorsal and ventral areas of the hippocampus.

Importantly, we found that the hippocampal part where some protein/peptide ligands, which are related to either “typical” signaling peptides or axon guiding cues, were expressed at higher level did not always coincide with the hippocampal part where their receptors were predominantly expressed. This splitting may point to the important role played by these signaling systems (semaphorins, slit-robo, ephrins) in the formation of longitudinal synaptic contacts (in case of axon guiding molecules) and information transfer along the longitudinal hippocampal axis.

An important aspect of our study is that we also analyzed the expression of cell-specific genes in the dorsal and ventral hippocampus. We found that expression of some markers of all major cell subpopulations, including neurons, astrocytes, oligodendrocytes, microglia, and vascular cells, differed between the studied hippocampal parts. The most numerous group of genes is related to oligodendrocytes. So, far it is not clear what determines a higher expression of oligodendrocyte-related genes in the dorsal hippocampus. Presumably, it may be related to a higher number of oligodendrocytes in this hippocampal part because a large number of myelinated axons, which originate from neurons along entire hippocampus, pass the dorsal part of the hippocampus to form the fimbria/fornix. Among the neuron-specific genes, one group of genes may be characterized as markers of interneurons (gad1, sst, vip, tac3, dlx1) and a dorsoventral gradient of their expression may reflect the potential differences in the number (and/or size) of the specific subpopulations of interneurons in the dorsal and ventral hippocampus. This difference in the number of interneurons expressing some markers was demonstrated in [25], and our data were in agreement with it in respect to the gradient of sst expression. Interestingly, practically all previous studies focused on hippocampal interneurons have never considered a potential role that the relationship between the distinct subpopulations of interneurons may play in the functioning of the dorsal and ventral hippocampus.

The least numerous groups of cell-specific genes differentially expressed between the hippocampal parts were related to astrocytes, microglia, and vascular cells. The differences in the number of cells related to these subpopulations have not been described so far and the small number of cell-specific markers suggests that the difference in the number of cells hardly explains the observed differences but rather reflects shifts in some characteristics of the cells in one of the hippocampal parts. The role of these gradients in the functioning of the dorsal and ventral areas of the hippocampus remains unclear and requires further study.

### 3.3. Expression of Transposable Elements

We found that both parts of the hippocampus expressed all the major types of transposons, which is in agreement with previous reports (reviewed in [18]). We showed here that the analysis of TE expression is better performed after the depletion of ribosomal RNA than after polyA-enrichment pointing to the fact that TE of all types expressed in both hippocampal parts were mostly not polyadenylated. Our data suggest that the amount of LINE, LTR, and SINE elements strongly increased in the RBD samples compared to the polyA-selection. Moreover, we showed that the majority of the transcribed intergenic, sense, and antisense intronic TE were not polyadenylated and this seems to be a rule for LINE elements L1 and L2, endogenous retroviruses, Alu elements, etc., in both hippocampal parts. One of the unusual findings that we made during our analysis was that TE were almost absent in transcripts related to 5′UTRs compared to the other parts of annotated genes. Presumably, this is related to the considerably shorter sequence of 5′UTR compared to the coding sequence and 3′UTR, although we cannot exclude the existence of some additional regulatory mechanisms that may suppress TE transcription from 5′UTRs.

We also show that the expression of the majority of TE was weakly shifted to the ventral hippocampus and this shift partly coincided with the dorsoventral differences that we found in previously published data. Moreover, our analysis of the previous developmental data points to the possibility that the expression of TEs alters during maturation of the hippocampus and the ontogenetic changes also depends on the hippocampal part. However, due to the low coverage and small number of samples, it is hard to make an unambiguous conclusion about ontogenetic changes and further targeted studies are necessary to clarify the importance of these changes.

## 4. Materials and Methods

### 4.1. Animals

All experimental procedures were conducted in accordance with the European Communities Council Directive of 24 November 1986 (86/609/EEC) on the protection of animals used for scientific purposes. The study protocol was approved by the Ethics Committee of the Institute of Higher Nervous Activity and Neurophysiology of RAS (approval number: 2019-012).

### 4.2. Sample Acquisition for RNA-seq

Wistar male rats aged 3 months were used for this study. The rats were handled for 4 days before tissue collection. The animals were decapitated, and hippocampi were isolated. The hippocampus, including the dentate gyrus, CA3, CA2, CA1, and subiculum, was equally trisected to divide it into the ventral, intermediate, and dorsal parts. Only the ventral and dorsal parts were collected for the study, frozen in liquid nitrogen and then stored at −70 °C until RNA isolation.

### 4.3. RNA Extraction, cDNA Library Construction, RNA-seq and Data Analysis

Total RNA for polyA-enrichment was extracted using Qiazol reagent (Qiagen, Hilden, Germany) according to manufacturer’s protocol. For RNA sequencing, libraries were prepared according to the following protocol: (1) mRNA isolation from total RNA with oligo(dT)-coated magnetic beads; (2) RNA fragmentation; (3) cDNA generation by reverse transcription using random primers followed by double-stranded cDNA synthesis; (4) reparation of ends, 3′-adenylation, and ligation of adapters; (5) polymerase chain reaction using adapters (PCR products were purified with Ampure XP Beads and dissolved in EB solution); (6) the library was validated with an Agilent Technologies 2100 bioanalyzer; (7) the double-stranded PCR product was denatured and cyclic molecules were synthesized from it using the splint oligo sequence, and the result was a library of single-stranded cyclic molecules; (8) the library was amplified with phi29 DNA polymerase to form nanodroplets containing more than 300 copies of a single DNA molecule. Sequencing was performed on a DNBseq platform in the “Genomed” company (Russia).

Total RNA for samples undergoing depletion of ribosomal RNA was extracted using ExtractRNA reagent (Evrogen, Russia) following the manufacturer’s protocol. Depletion of ribosomal RNA was performed using a MGIEasy rRNA Depletion kit. Sequencing was performed on the DNBseq-G400 platform in the “Genomed” company (Russia).

### 4.4. Bioinformatic Analysis

#### 4.4.1. Read Mapping and Counting

To estimate gene expression, the sequenced reads were mapped to the ENSEMBL version of Rat genome (Rnor6.0) using STAR [26] with default parameters and raw read counts obtained using FeatureCounts [27]. For each dataset, counts were normalized by the “median-of-ratios” method and subjected to differential expression analysis using DESeq2 R-package [28] with chosen *p.* adjusted significance level < 0.1. To estimate the transposable element expression, reads were re-mapped using STAR with parameters, allowing for multimapping reads detection—*outFilterMultimapNmax 500* and—*winAnchorMultimapNmax 500*. We used RepeatMasker rn6 genome annotation, included in the TEtranscripts software [29] as a reference for counting and loci-level counted TE with the Bayesian mixture model approach, implemented in Telescope [30]. Estimation of TE loci position within genes (CDS, UTR) was made with custom scripts and GenomicRanges R-package [31], and family-level differential expression TE analysis was performed using DESeq2 while utilizing per-sample normalization (size) factors from gene DE. For estimation of transcript abundance, we used Salmon [32] and assessed differential transcript usage in both datasets through the DEXSeq-StageR pipeline [21] with StageR provided *p*. adjusted < 0.1. Expression per all non-overlapping exons in each gene (“exon bins”) was counted with DEXSeq scripts prepare_annotation.py and dexseq_count.py [33].

#### 4.4.2. Gene Ontology and Visualisation

Gene ontology/KEGG category search was performed using the clusterProfiler R-package [34], with a chosen *p*. adjusted significance level < 0.05. KEGG pathways were visualized with the Pathview [35] internet resource. Gene expression visualizations were carried out using R-packages ggplot2, ComplexHeatmap [36], VennDiagramm [37]. Regularized logarithm expression values were used for constructing gene- and family-level TE heatmaps. Transcript model images were constructed by IsoformSwitchAnalyzeR package [38].

#### 4.4.3. RNA-seq Data Access

RNA-seq data was deposited in the National Center for Biotechnology Information Gene Expression Omnibus (GEO) under the accession number GSE208330; a link for reviewers: https://www.ncbi.nlm.nih.gov/geo/query/acc.cgi?acc=GSE208330 (accessed on 28 August 2022).

## 5. Conclusions

Previous analysis of the functions of the dorsal and ventral parts of the hippocampus suggests that these hippocampal parts are involved in the implementation of different functions despite a similar histological structure. We further extended these data and showed that different technical approaches revealed different specific features of gene expression in the hippocampal parts. We compared the data obtained using typical polyA-enrichment with the results of the analysis of the entire RNA pool comprising both polyadenylated and non-polyadenylated RNA. The data on the differential expression of mRNA largely coincided between datasets obtained using different approaches whereas the results of the splice isoforms and transposable elements strongly differed. The detected differences point to the necessity of the generation of common approaches for the analysis of gene expression in the hippocampus, but our data suggest that analysis of the expression of transposable elements should be performed in samples after depletion of ribosomal RNA.

We showed that the functional splitting of the hippocampus was associated with the predominant expression of genes related to various neurotransmission cascades and proteins involved in intercellular communication in one of the hippocampal parts. Moreover, our analysis of the expression of transposable elements, which serve as regulators of transcription and translation machinery, revealed a slightly higher expression of the majority of the transposable elements in the ventral hippocampus pointing to the possible basis of differential gene expression between the hippocampal parts.

Our analysis based on the expression of cell-specific markers showed that the dissimilarity between the hippocampal parts also included differences in the expression of several astrocytic, microglial, and vascular markers pointing to the possibility that the functions of these cells may differ between the hippocampal subdivisions. This, as well as the putative gradient in the density of oligodendrocytes and interneurons between the dorsal and ventral parts, provided an additional dimension to the structural heterogeneity of the hippocampal parts.

## Data Availability

RNA-seq data was deposited in the National Center for Biotechnology Information Gene Expression Omnibus (GEO) under the accession number GSE208330; a link for reviewers: https://www.ncbi.nlm.nih.gov/geo/query/acc.cgi?acc=GSE208330 (accessed on 28 August 2022).

## Supplementary Materials

The following supporting information can be downloaded at: https://www.mdpi.com/article/10.3390/ijms23179948/s1.
