# Supplementary material for: Detailed Analysis of Dorsal-Ventral Gradients of Gene Expression in the Hippocampus of Adult Rats
Source: Int J Mol Sci. 2022 Sep 1;23(17):9948. doi: 10.3390/ijms23179948 (PMC9456131; doi:10.3390/ijms23179948)
Supplement: Supplementary file 1 [file ijms-23-09948-s001.zip › ijms-1848265-supplementary.pdf]

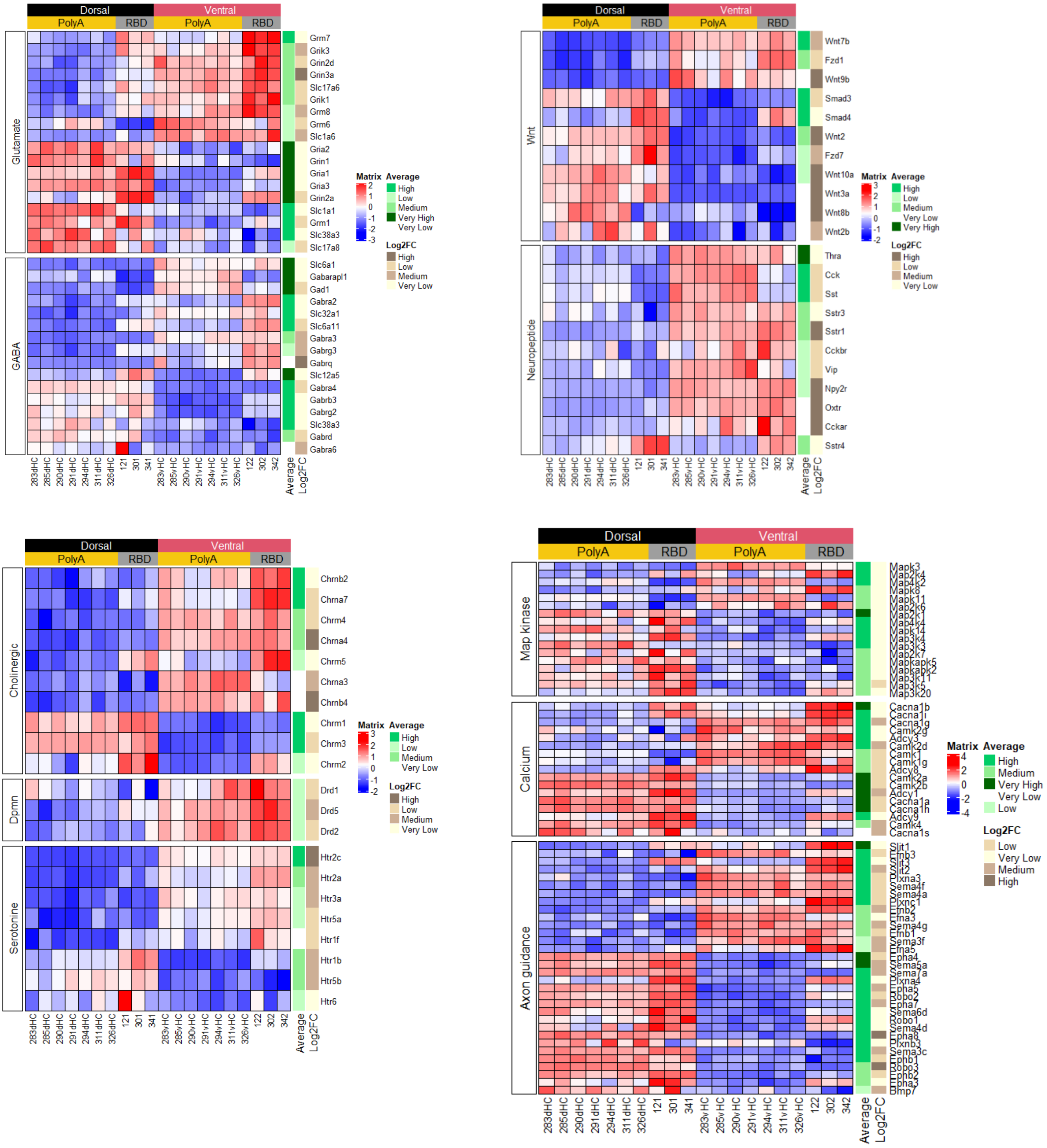

Figure S1. Extended heatmaps showing all DE genes for KEGG categories, related to neurotransmitters, calcium signaling and axon guidance cues. All designations are identical to Fig 2. in the main text.

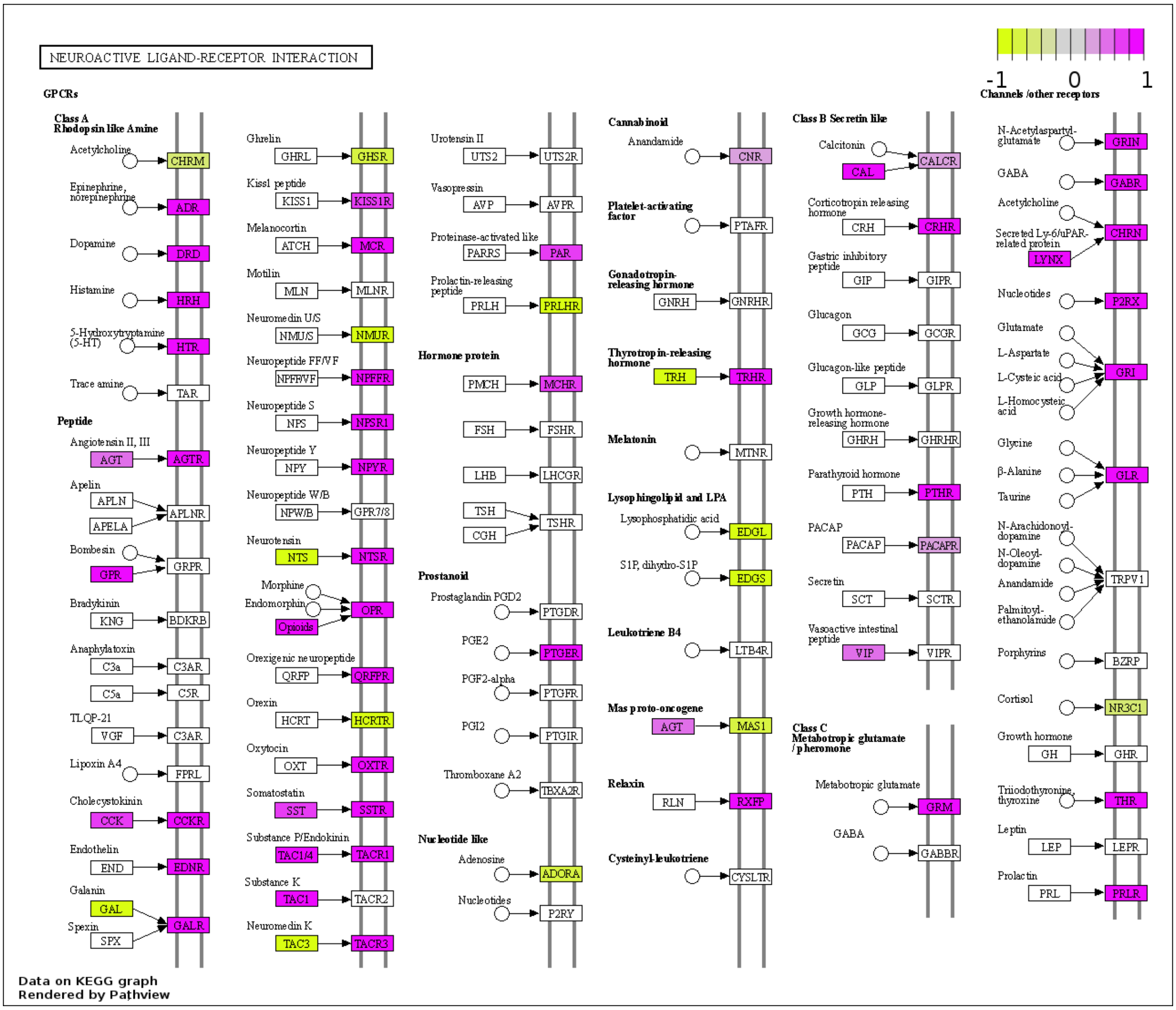

Figure S2. Visualisation of Neuroactive ligand-receptor interaction KEGG pathway. Dorsal-enriched DE genes are shown in yellow; ventral, in purple.

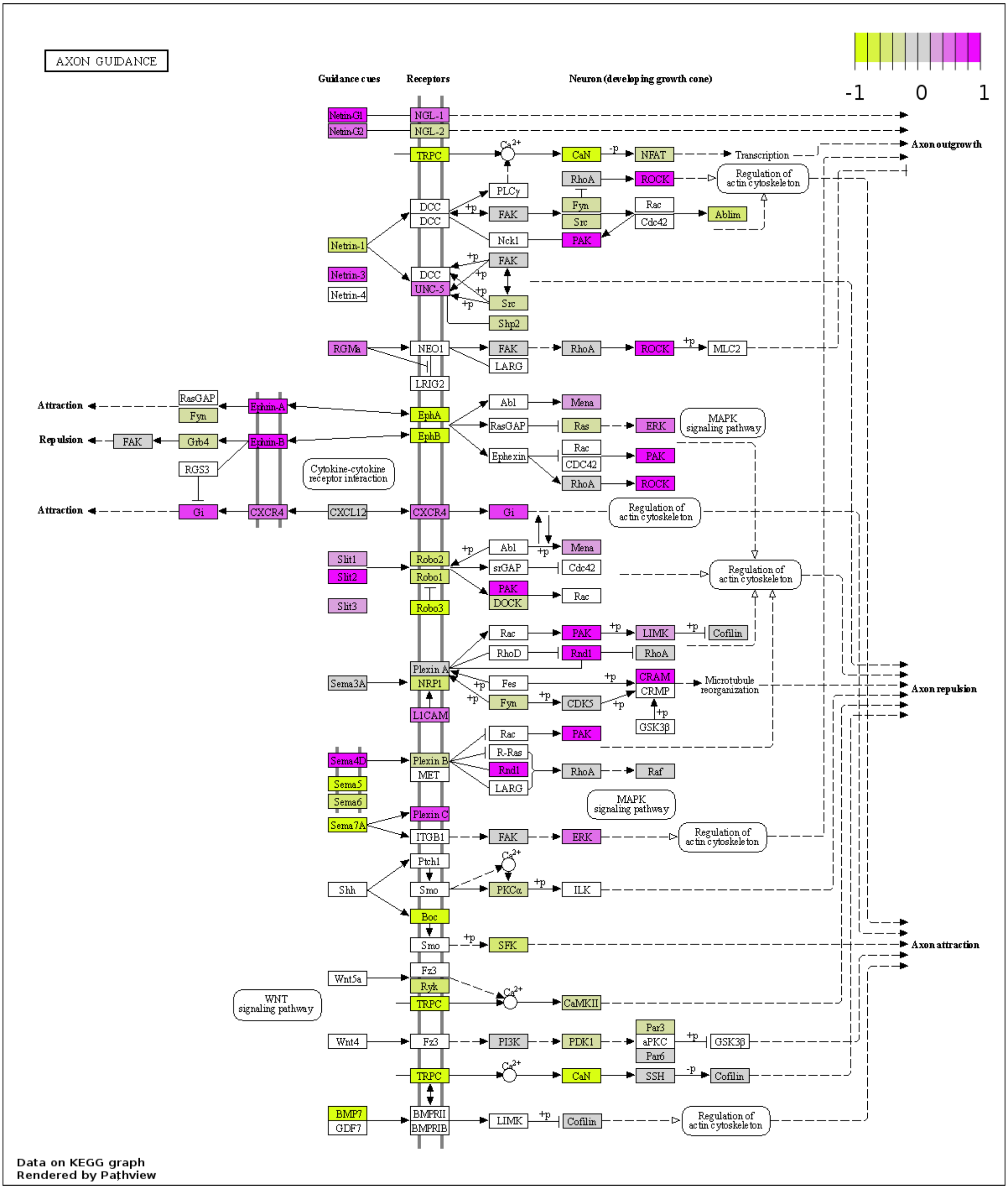

Figure S3 .Visualization of axon guidance signaling KEGG pathway. Dorsal-enriched DE genes are shown in yellow, Ventral - in purple.

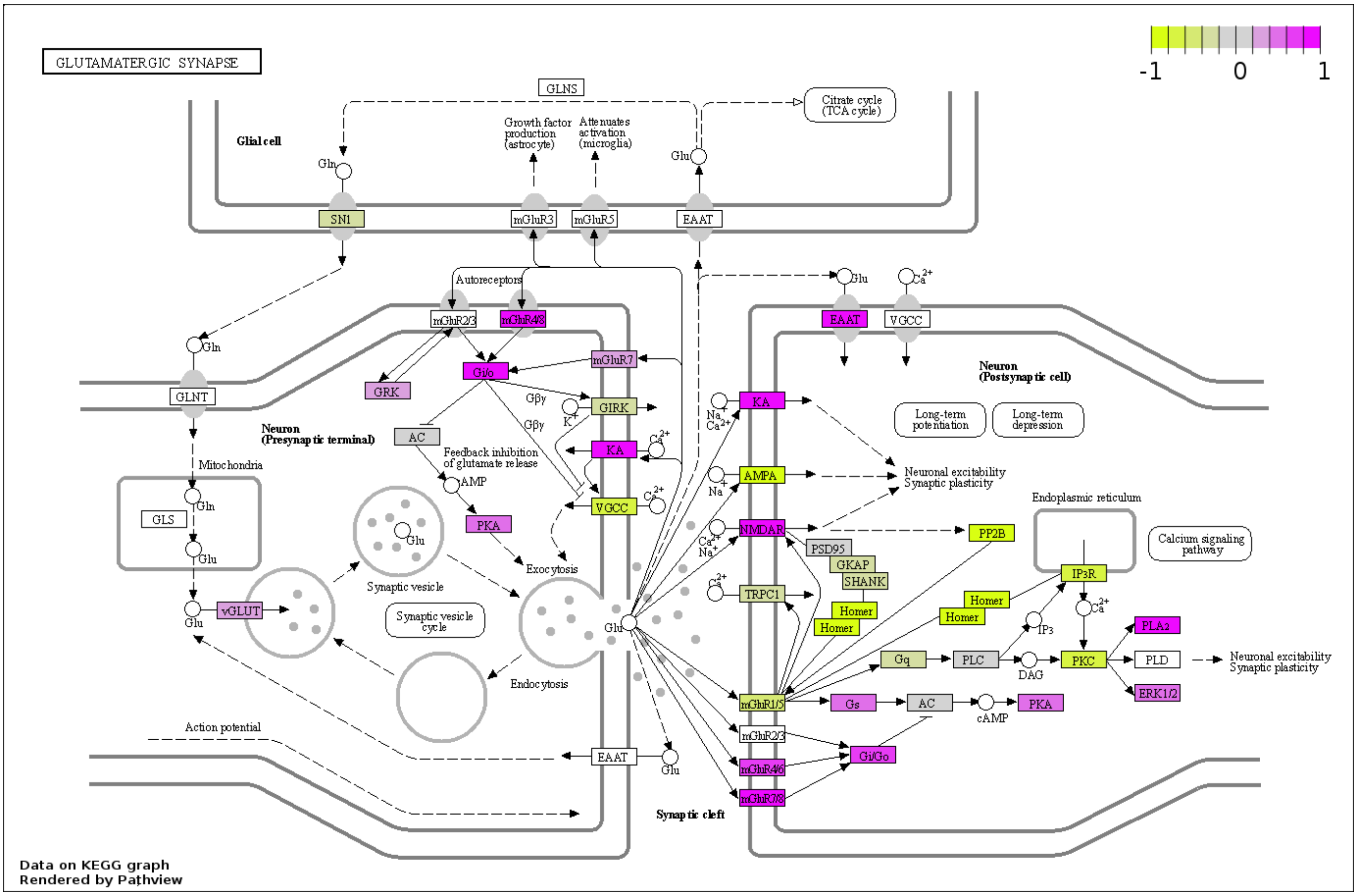

Figure S4.Visualisation of Glutamate-related signaling KEGG pathway. Dorsal-enriched DE genes are shown in yellow; ventral, in purple.

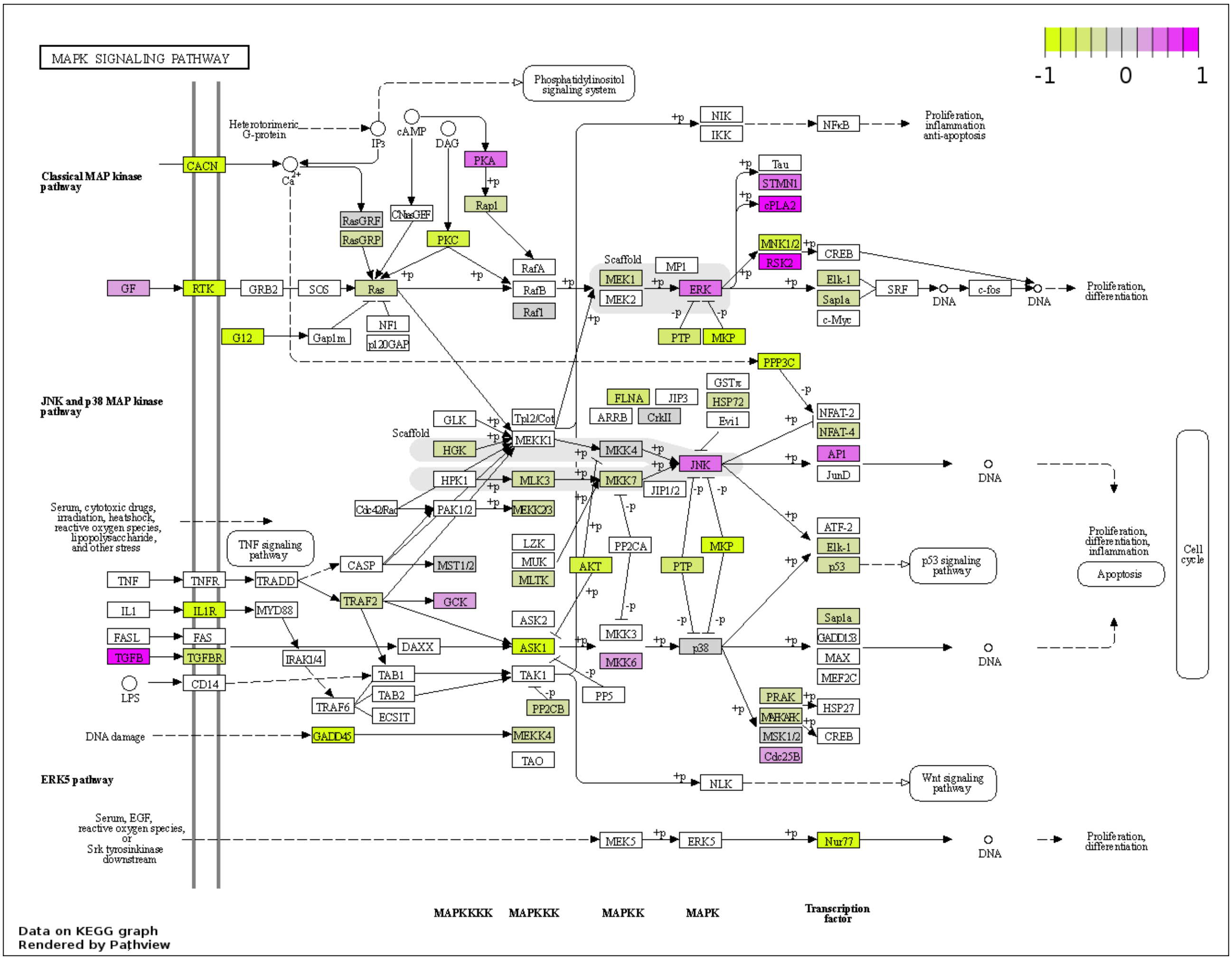

Figure S5 .Visualization of MAPK signaling KEGG pathway. Dorsal-enriched DE genes are shown in yellow, Ventral - in purple.

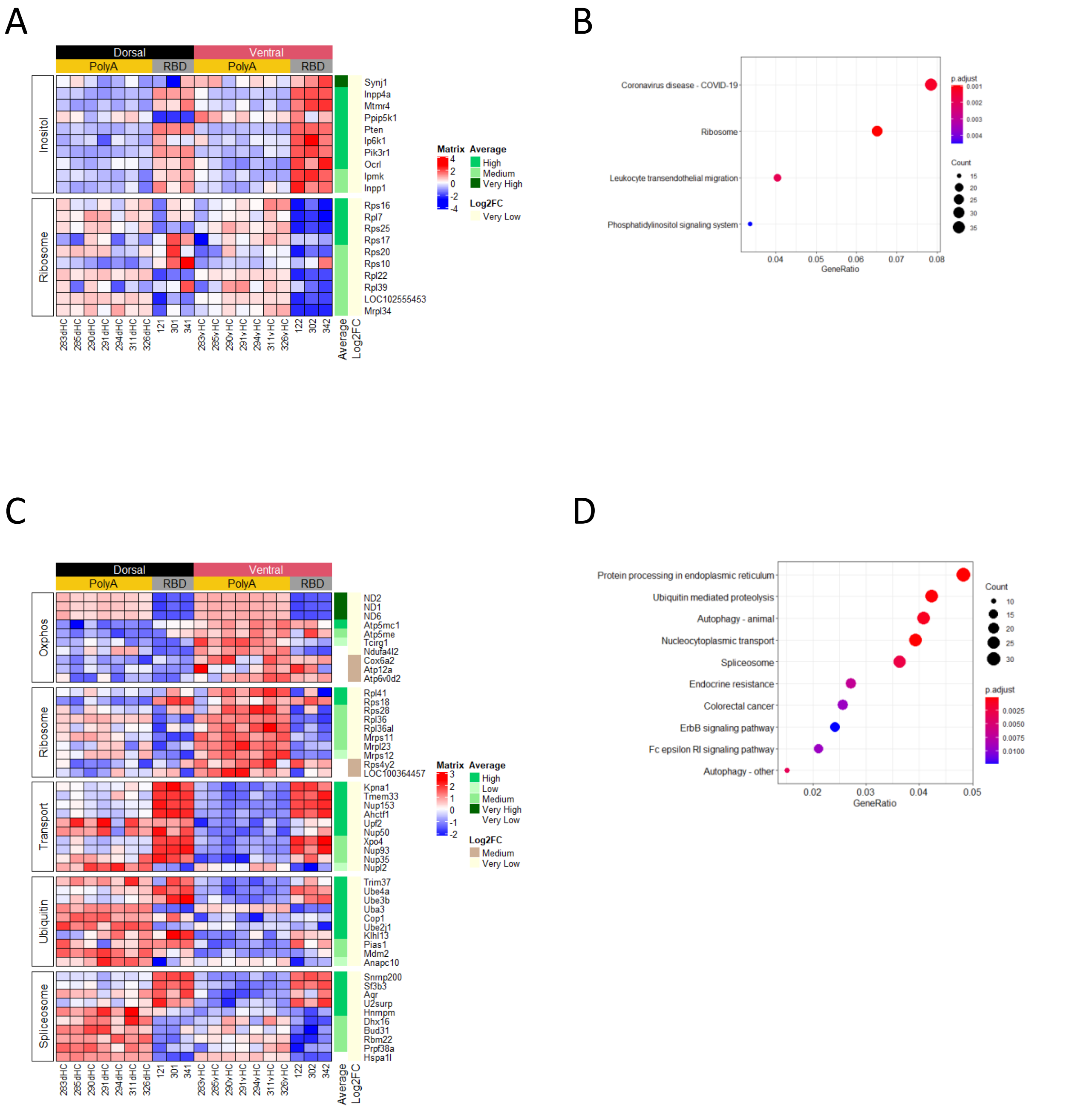

Figure S6 (A) Heatmap and (B) KEGG categories plot for RBD specific DE genes. (C) Heatmap and (D) KEGG categories plots (upper - VH, lower - DH) for PolyA specific DE genes.

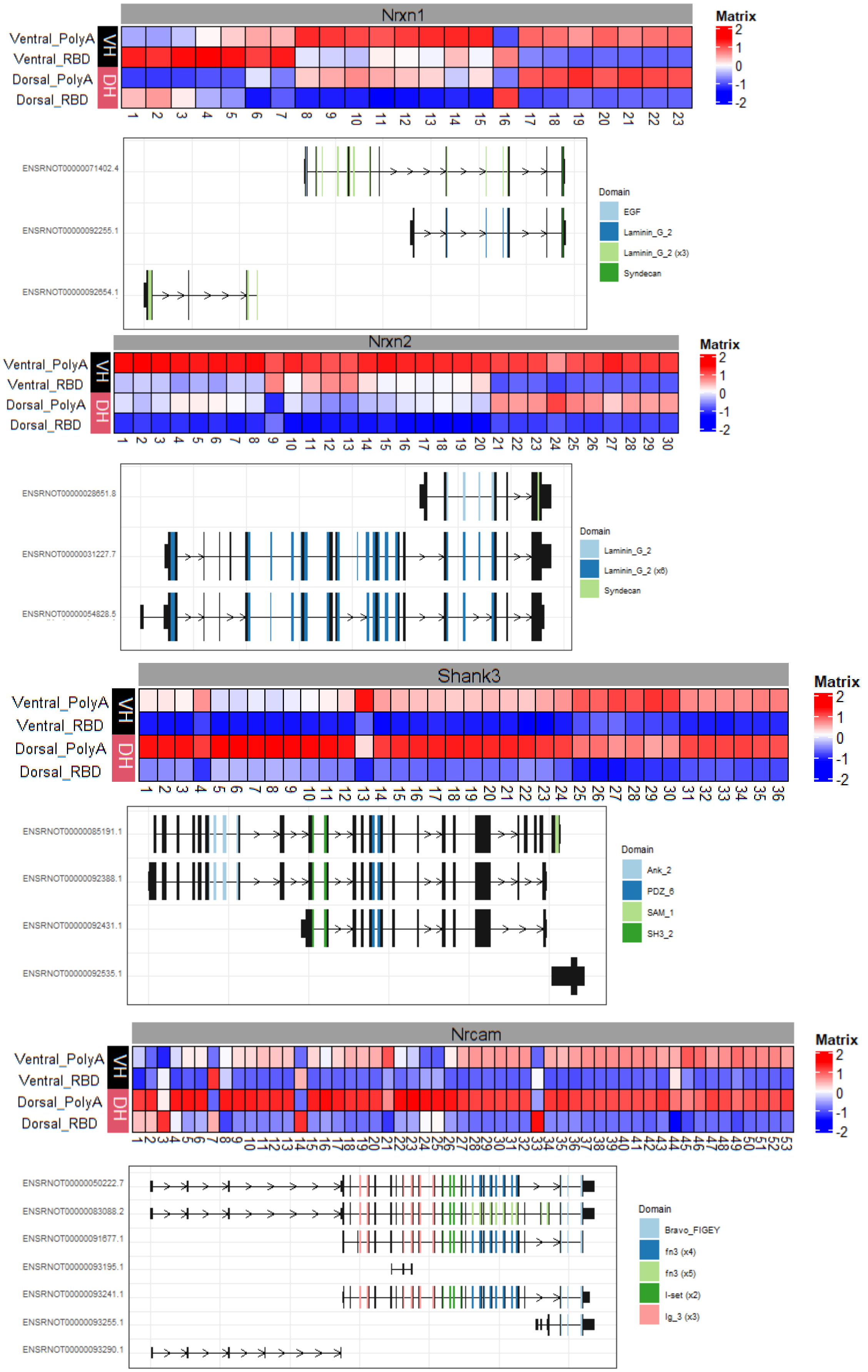

Figure S7. Example heatmaps showing expression level of non-overlapping exon bins (Anders et al., 2012) for certain genes from DTU list which roughly correspond to their trancript models which are shown below each gene. Exon bin counts are averaged across all samples from relevant dataset and z-scored per exon bin.

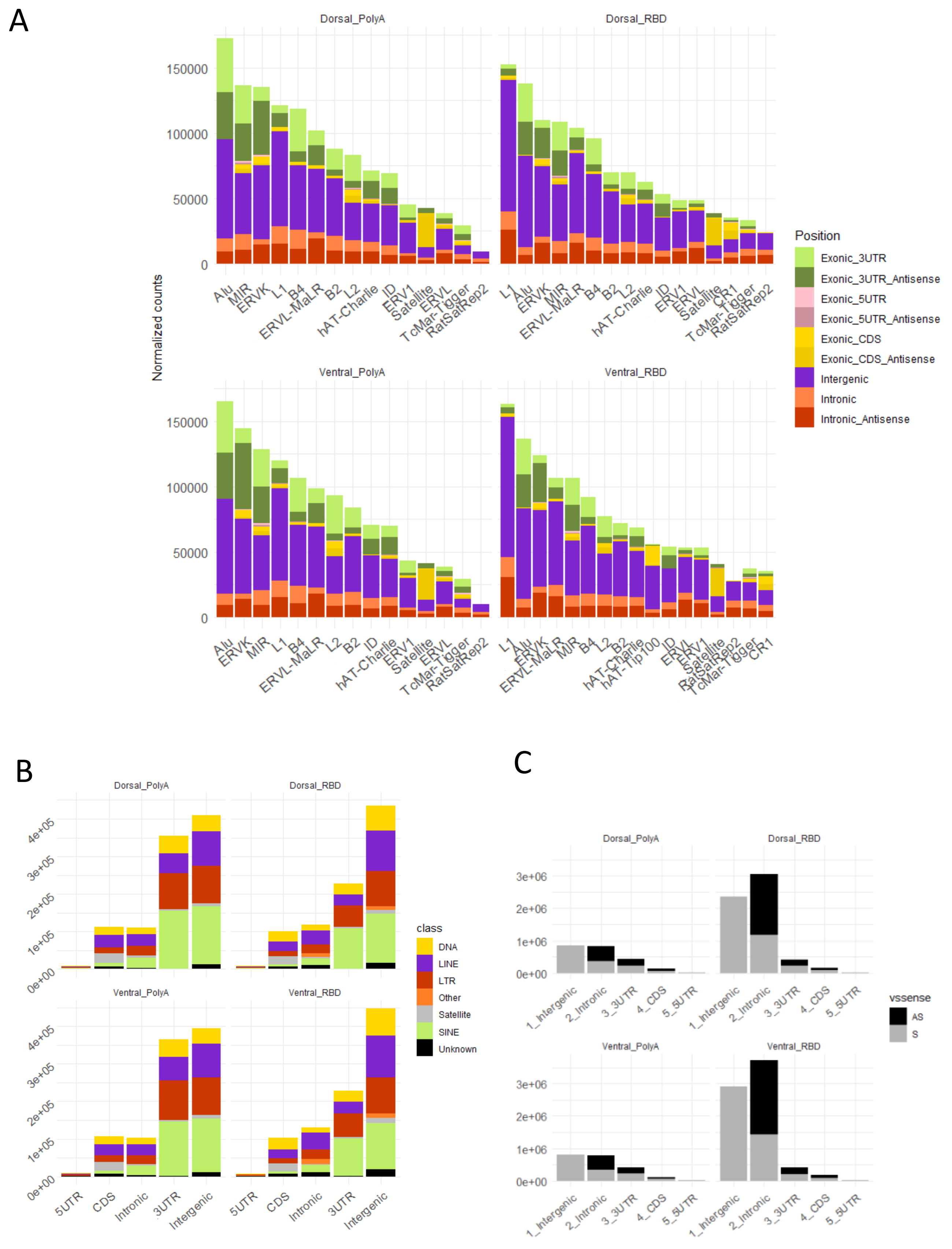

Figure S8. (A) Normalized reads count per 1000 top expressed loci of each repeat family, summarized by different genic/intergenic position. (B) Normalized reads count per genic/genomic position for 1000 top expressed loci per family, summarized by repeat class. (C) Normalized reads count per genic/genomic position for all loci summarized by strandedness.

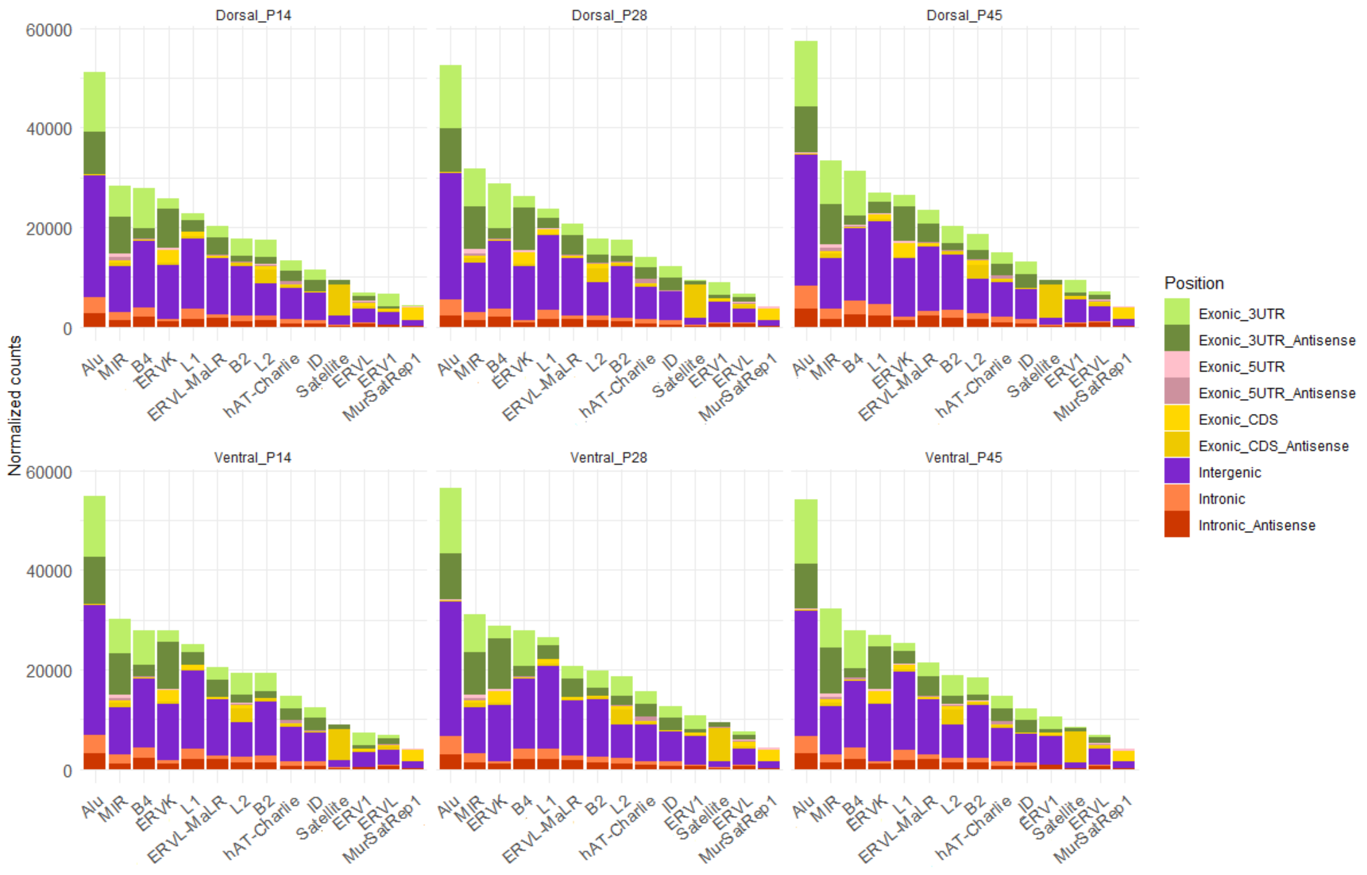

Figure S9. Normalized reads count per repeat family for developlmental dataset summarised by number of different genic/intergenic positioned loci in each.
